# Aesthetic Plastic Surgery in India Is Poised for Exponential Growth and Global Dominance

**DOI:** 10.1055/s-0044-1800876

**Published:** 2024-12-27

**Authors:** Dinesh Kadam

**Affiliations:** 1 Department of Plastic and Reconstructive Surgery, A.J. Institute of Medical Sciences and Research Centre, Mangalore, Karnataka, India

**Fig. 1:**
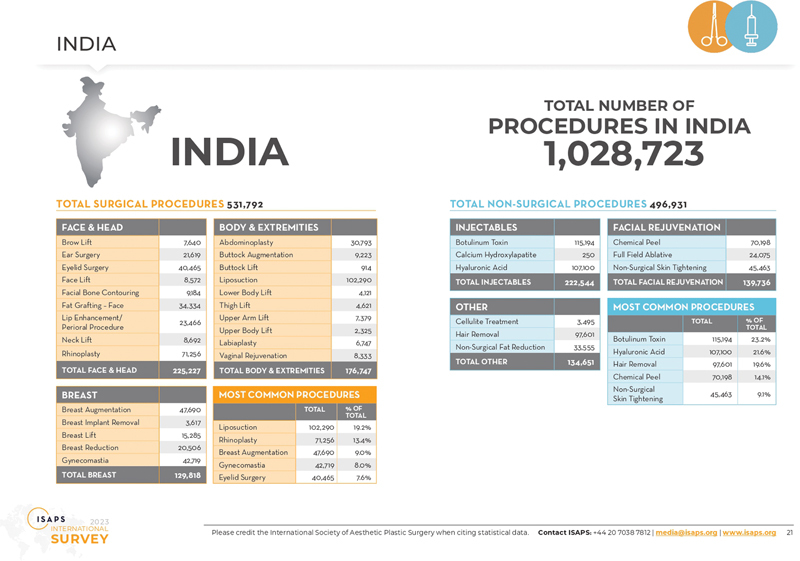
Total aesthetic procedures performed in India in 2023. (This image is provided courtesy of the International Society of Aesthetic Plastic Surgery Global Survey, 2023.)

The 2023 ISAPS Global Survey suggests India is ranked second and third in rhinoplasty and liposuction surgeries and seventh overall in surgical and nonsurgical aesthetic procedures. Further, India ranks second in nonsurgical facial rejuvenation done by plastic surgeons. 1 With over 1 million aesthetic procedures in a year by plastic surgeons, India has taken a giant leap with an upward growth trajectory ( Fig. 1 ).

Aesthetic surgery in India has seen exponential growth thanks to rising disposable incomes, increased awareness of cosmetic procedures, improved availability of expertise, and medical tourism. According to recent reports, the cosmetic surgery market in India is expected to grow at a compound annual growth rate of 10 to 12% over the next 5 years. 2 In terms of revenue, India accounted for 7.4% of the global aesthetic surgery procedures market in 2023. The revenue generated by the Indian cosmetic surgery procedure market was 4,198.2 million USD in 2023, and it is predicted to reach 11,567.3 million by 2030. The United States is expected to have a dominant revenue position in the global market by 2030. India is the fastest-growing regional market in Asia Pacific and is projected to lead the regional market in terms of revenue in 2030.

With over 2,000 plastic surgeons catering mainly to urban India, the potential for reaching rural and remote areas remains a challenge. The rapid evolution of aesthetic plastic surgery in tier I and II cities are causing plastic surgeons to encounter new challenges, as well as fierce competition among their colleagues in plastic surgery. A multitude of nonplastic surgery practitioners have entered fields traditionally served by plastic surgeons. These challenges are compounded by the exponential increase in promotion and marketing strategies by nonplastic surgeons on social media platforms. Apparently, the days of relying on physician referrals, word of mouth, academic pedigrees, and achievements for practice building in plastic surgery are long gone. There is no denying that electronic platforms and social media are here to stay as we are amid an unstoppable shift in social mindset and consciousness. Patients are relying more on social media for advice and decision-making regarding plastic surgery procedures. 3 Adhering to ethical and scientific principles and respecting clients' privacy concerns, one can steadily maintain an online presence, remain relevant, and build up ethical practice.

In a busy aesthetic surgery private practice, focusing on research and publications often takes a low priority. Nevertheless, academic growth through publications plays a pivotal role in the career and reputation of plastic surgeons. Essentially, there are several benefits. Engaging in academic work pushes us to stay updated on the latest techniques, technologies, and trends, promoting evidence-based practice. Publishing in peer-reviewed journals enhances the credibility and reputation within the medical community, and they often gain leadership roles in professional organizations and societies. Increased visibility brings opportunities for international collaborations and invitations to contribute to reputed meetings. Surgeons with credible publication records can mentor trainees and junior colleagues, fostering a culture of research. A strong academic profile also gives patients confidence in the surgeon's expertise and reliability, particularly in the competitive aesthetic surgery market. Patients seeking high-quality care are more inclined to prefer surgeons who contribute to academic advancements.

While Indian plastic surgeons are breaking barriers in the international community and contributing with a record number of clinical work, it is even more imperative that we promote scientific publications to enhance credibility and command authority. In this regard, we solicited manuscripts on aesthetic plastic surgery earlier this year. I am delighted to bring you this *special issue of IJPS* dedicated to aesthetic plastic surgery. I express my heartfelt appreciation and admiration to all the authors for their contributions. I am humbled by my reviewers for their continued support of the journal, and I am indebted to them.

The growth of aesthetic plastic surgery in the country is exponential. If we all make small steps toward research and publication, India's leading position in aesthetic surgery can be significantly enhanced.
